# Finite element modeling of plasmonic resonances in photothermal gold nanoparticles embedded in cells†

**DOI:** 10.1039/d4na00247d

**Published:** 2024-07-10

**Authors:** Marina París Ogáyar, Rosalía López-Méndez, Ignacio Figueruelo-Campanero, Tamara Muñoz-Ortiz, Claire Wilhelm, Daniel Jaque, Ana Espinosa, Aida Serrano

**Affiliations:** a Instituto de Cerámica y Vidrio (ICV-CSIC) C/Kelsen, 5 Madrid 28049 Spain aida.serrano@icv.csic.es; b IMDEA Nanociencia C/Faraday, 9 Madrid 28049 Spain; c Nanomaterials for Bioimaging Group (nanoBIG), Departamento de Física de Materiales–Facultad de Ciencias, Universidad Autónoma de Madrid C/ Francisco Tomás y Valiente, 7 Madrid 28049 Spain; d Departamento de Física de Materiales, Universidad Complutense de Madrid Plaza Ciencias, 1 Madrid 28040 Spain; e Instituto Nicolás Cabrera, Facultad de Ciencias, Universidad Autónoma de Madrid C/ Francisco Tomás y Valiente, 7 Madrid 28049 Spain; f Laboratoire Physico Chimie Curie, PCC, CNRS UMR168, Institut Curie, Sorbonne University, PSL University Paris 75005 France; g Institute for Advanced Research in Chemical Sciences (IAdChem), Universidad Autónoma de Madrid C/Francisco Tomás y Valiente, 7 Madrid 28049 Spain; h Instituto de Ciencia de Materiales de Madrid (ICMM-CSIC) C/ Sor Juana Inés de la Cruz, 3 Madrid 28049 Spain ana.espinosa@csic.es

## Abstract

The use of plasmonic nanoparticles in performing photothermal treatments in cancer cells requires a full knowledge about their optical properties. The surface plasmon resonance is easily foreseen and measurable in colloidal suspensions, however it can be strongly modified when located inside cells. Assessing the optical behavior of plasmonic nanoparticles in cells is essential for an efficient and controlled treatment. This requires the combination of experimental data and computational models to understand the mechanisms that cause the change in their optical response. In this work, we investigate the plasmonic response of Au nanospheres (AuNSs) internalized into cancer cells (MCF-7). Experimental data are compared to the simulations provided by a 3D model based on a finite element method. We demonstrate the impact of physical parameters such as the type of NS assembly, the surrounding medium and the interparticle gap, in the photothermal efficiency of AuNSs. Results open the avenue to predict, by numerical calculations, the optical properties of plasmonic nanoparticles inside cells to minimize treatment costs and times in photothermal therapies.

## Abstract 2

The plasmonic response of Au nanospheres (NSs) in MCF-7 cells is investigated comparing experimental data with 3D simulations. Our findings highlight the impact of NS assembly, the surrounding medium, and interparticle gap on photothermal efficiency.

## Graphical abstract

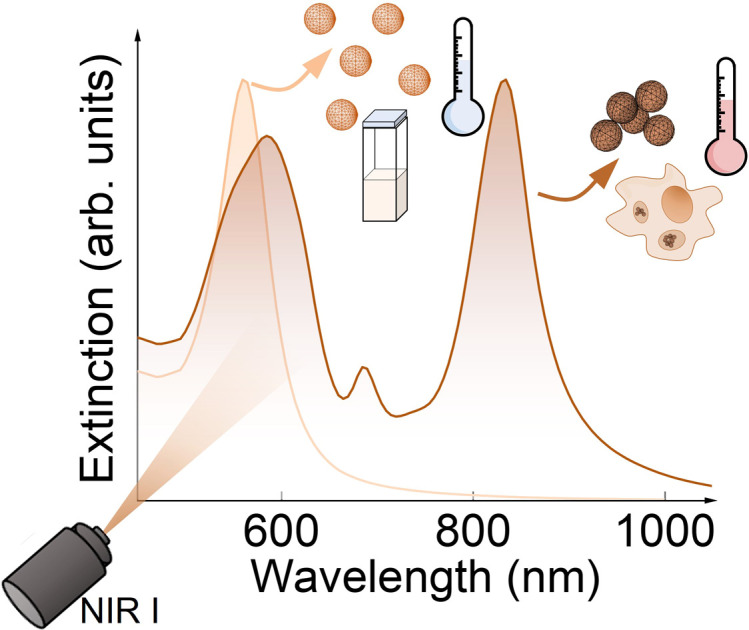

## 1 Introduction

Noble metal nanoparticles (NPs) such as gold (Au), silver (Ag) or platinum (Pt) exhibit very attractive optical and thermal properties for multiple biomedical applications.^1,2^ Specifically, Au nanoparticles (AuNPs) stand as one of the most interesting nanostructures for diagnosis and therapies due to their unique features such as the intense localized surface plasmon resonance (LSPR),^3^ optical tunable properties and high biocompatibility.^4^ Some promising AuNPs display their LSPR within the UV-visible spectral range, which limits their efficacy in specific biomedical applications due to the reduced penetration of light in biological tissues within this spectral range.^5,6^ In order to effectively treat deeply embedded tumors, it is crucial for nanostructures to exhibit efficient light-to-heat conversion capabilities in the near-infrared (NIR) region, either in the first biological window (NIR-I, *λ* = 650–950 nm) or in the second one (NIR-II, *λ* = 1000–1350 nm).^7–9^

The influence of intrinsic parameters, such as size, shape, or composition, on the plasmonic response of metallic NPs is widely known,^3,10,11^ allowing the tuning of LSPR and shifting its position to NIR-I or NIR-II biological windows. Similarly, the plasmonic response of AuNPs can be affected by extrinsic variables, such as the physical surrounding environment.^12^ Indeed, dielectric properties associated with medium exchange may shift the characteristic absorption bands of AuNPs.^3^ Additionally, the aggregation of plasmonic NPs^13^ and the interparticle distance when AuNPs are close enough (<2 nm)^14^ affect significantly the optical signal. In both cases, the absorption spectral profile changes due to the induction of plasmon coupling leading to a redshift of the plasmon resonance and the appearance of secondary optical modes (*i.e.*, new bands).

Interestingly, among different AuNPs, spherical AuNPs or Au nanospheres (AuNSs) show distinctive features besides their plasmonic properties. AuNSs can be easily synthesized with well-established simple methods and exhibit lower cytotoxicity compared with surfactant cetyltrimethylammonium bromide (CTAB)-assisted syntheses employed for other AuNP geometries.^15^ Additionally, AuNSs can be easily functionalized with biomolecules such as peptides, proteins, polymers, drugs, or antibodies. Due to their efficient LSPR as well as the above-mentioned features, AuNSs can be used as exceptional nanoheaters for photothermal therapy (PTT) when they are subjected to laser light.^16^

Aggregation effects of metallic nanomaterials in cells and consequent modifications of their plasmonic response have already been reported experimentally, resulting in enhanced and beneficial photothermal applications.^8,17,18^ These effects are mainly due to rearrangements of the NPs inside the cells after intracellular confinement within lysosomes. Additionally, other contributions arise from differences in the ionic strength of the surrounding environment, which may also promote aggregation and impact electrostatic interactions.^19,20^ Theoretical studies have been reported for plasmonic response coupling of nanomaterials in aqueous media or arrays in substrates,^1,21–26^ which were corroborated with experimental measurements for some specific cases. However, further theoretical investigation is crucial to explore and predict the potential photothermal applications of these plasmon-induced phenomena within the cellular environment. Effective thermal treatments rely on optimizing the laser radiation wavelength with the plasmon resonance. This is because efficient heat generation requires high overlap between the two. However, different interactions between NPs and cells may lead to plasmon shift and weakening, resulting in insufficient heat generation. Therefore, it is important to conduct critical studies that evaluate the several intrinsic and extrinsic parameters involved in these interactions.

In this context, COMSOL Multiphysics software is a valuable tool that enables the simulation of optical absorbance of plasmonic nanostructures, being able to control different variables that may influence the final plasmonic response. Even though multiple simulations have been run to emulate LSPRs with theoretical approaches^27,28^ using computational methods such as discrete dipole approximation (DDA),^29^ finite difference in the time domain method (FDTD)^30,31^ and the boundary element method (BEM),^32,33^ none of them has addressed the aggregation state of the NPs in cell media. Investigations in this line will allow for the prediction of the experimental optical response, thereby enhancing the efficiency in specific applications.

This work presents a methodology based on a finite element method (FEM) using COMSOL Multiphysics to assess the involved physical parameters of NPs in the cellular context to be used as PTT agents and predict their response. Specifically, we evaluate AuNSs with different diameter sizes, internalized into breast cancer cells. The modeling using COMSOL Multiphysics is validated with Mie theory^34^ and experimental results. The evaluation involves a randomly oriented aggregate (mimicking the lysosome-like assembly) and several changes, not only in the aggregation state, but the optical properties of the surrounding media and the gap between the AuNSs. The emergent resonant modes resulting from the endosomal aggregation of AuNSs in cells after internalization are then associated with their photothermal performance under NIR-I light exposure.

## 2 Results and discussion

### 2.1 Verification and validation of the model using the finite element method (FEM)

**Fig. 1:**
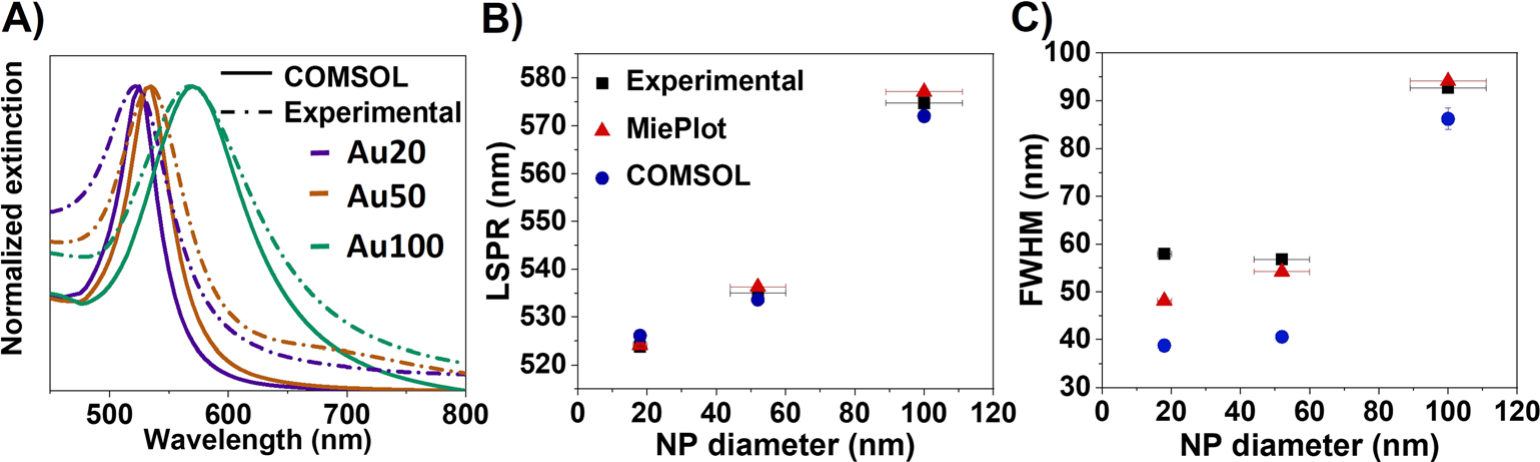
Comparison of the plasmonic response of AuNSs dispersed in water. (A) FEM simulation *vs.* experimental results. (B) LSPR position and (C) FWHM obtained by FEM and MiePlot simulations along with the experimental results. The *x*-axis error bars represent the polydispersity of the particles extracted from TEM analysis. AuNSs of 20, 50 and 100 nm in diameter were used in FEM simulations.

Fig. 1A displays the experimental optical extinction of AuNSs with different sizes (18 ± 2 nm (Au20), 56 ± 8 nm (Au50) and 100 ± 11 nm (Au100), see Fig. S2†). As expected, AuNSs show a redshift in the extinction band when increasing the diameter size. In particular, the LSPR maxima are 523.9 ± 0.1 nm, 535.0 ± 0.1 nm and 574.7 ± 0.2 nm for AuNSs of Au20, Au50 and Au100, respectively. In addition, a broadening of the plasmonic resonance is observed, which increases significantly with NS diameter: 57.9 ± 0.3 nm, 56.8 ± 0.2 nm and 92.7 ± 0.7 nm for Au20, Au50 and Au100, respectively. Both dependencies are in accordance with previous studies.^3,10,35^

The plasmonic behavior of small dispersed AuNSs is well described in the literature.^34,36^ However, these studies are limited for understanding the electronic response when systems present more complex geometries and particle assemblies. Numerical solvers of differential equations such as FEMs with boundary conditions, can provide general solutions that enable researchers to study the scattering and absorption of electromagnetic radiation by particles. FEM-based simulations are particularly well-suited for modeling more complex geometries, as they allow researchers to tailor the model to the particular scattering problem at hand.^37,38^ For this reason, in this work we run FEM simulations in COMSOL Multiphysics software for the case of AuNSs as they are internalized into cells. To accomplish this, a computational model is developed using COMSOL. The model comprises a spherical 6-layer perfectly matched layer (PML), which depends on the size of the AuNSs, and has a size of at least 2 times the scattered dimensions^37^ (*i.e.*, size of AuNSs or AuNS aggregates).

Specific simulations with AuNSs in aqueous media were carried out with relative permittivity, electrical conductivity and relative permeability of *ε*_r_ = 1.7689, *σ* = 0 and *μ*_r_ = 1, respectively. For plasmonic response evaluation of AuNSs in cells, the relative permeability of the media was fixed to *ε*_r_ = 2.56 according to refractive index (*n*) measurements in lysosomes.^39^ For the dielectric function of AuNSs, data from Johnson and Christy were used in the simulations.^40^

In simulations, AuNSs are illuminated with circularly polarized light to mimic light dispersion through tissues. Simulating unpolarized light doubles the computing time but gives comparable results.^41^ Scattering and absorption cross-sections were evaluated by integrating the scattered Poynting flux vector (*S*_sca_) over the surface of the NPs and by calculating the volume integral of the power dissipation (*Q*) inside the NPs:^37^1
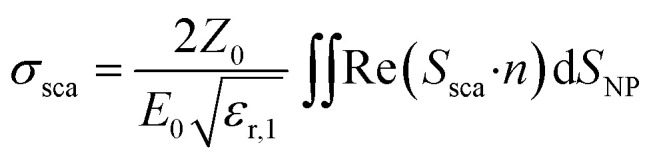
2
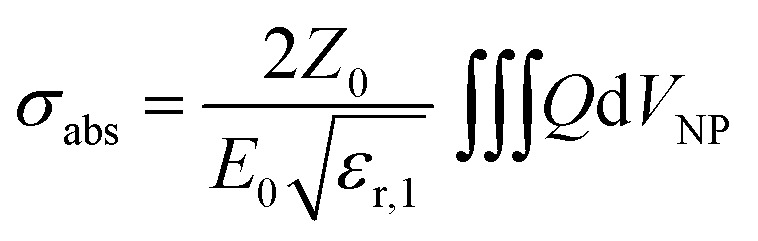
where *ε*_r,1_ is the relative permittivity of the surrounding environment, 
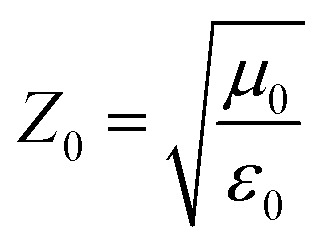
 the impedance of vacuum and *E*_0_ the amplitude of the incident wave.

Finally, extinction cross-section values were calculated as a sum of scattered and absorbed light by the incident wave. Calculated extinction curves are displayed in Fig. 1A along with the experimental data.

The developed FEM model was initially validated by comparing the experimental results obtained for different sizes of AuNSs in aqueous dispersion (water) with those obtained from the Mie theory. A good agreement can be observed for the different sizes of AuNSs, in terms of both the position and the full width at half maximum (FHWM). The average error between the computational method (FEM model), analytical method (MiePlot program) and the experiment is less than 1% for the resonance position (see Fig. 1B). However, it should be noted that for the FWHM (Fig. 1C), this error increases up to 30% in the FEM model due to model limitations (*i.e.*, when polydispersity of NPs is not considered), deriving in narrower bands than the ones observed experimentally. In fact, it is further noted that the difference between the numerical model and experiments increases for the smallest AuNSs (Au20), which is in good agreement with previous studies.^42,43^ This result is attributed to the fact that the dielectric functions measured by Johnson and Christy correspond to bulk materials,^44^ which leaves wider resonances in the experimental measurements than those of the numerical model.

**Fig. 2:**
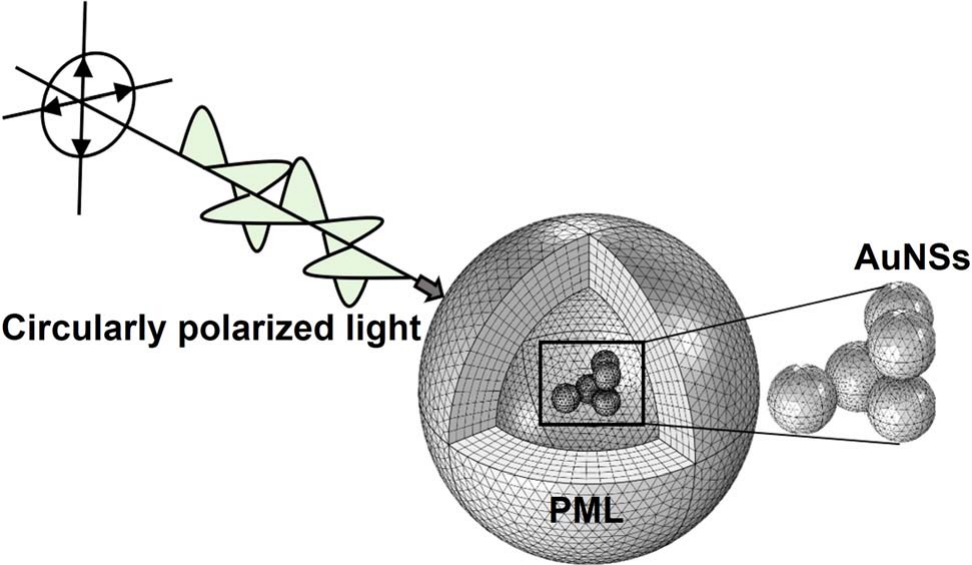
Example of a simulation by the FEM model developed. Structure of the model representing an aggregate of 5 AuNSs in cell media being illuminated by circularly polarized light. The PML is composed of 6 mesh elements across its thickness. The PML is dependent on the AuNS size, assuring that it is at least twice the maximum scattering dimensions.^37^

Once the developed FEM model has been validated, the 3D model was employed to evaluate the optical response when AuNSs are internalized into cells. To achieve this, different extinction-dependent scenarios were simulated according to the literature, evaluating the aggregation state of AuNSs as well as their interparticle gap and surrounding media.^45,46^ The simulation of plasmonic response controlling parameters such as the interparticle size and the aggregate state of NPs, as shown here using the validated FEM model, is not possible by developed softwares such as MiePlot. Fig. 2 shows an example of a model employed for the simulations with a randomly positioned aggregate as a representative example.

### 2.2 Internalization and cell uptake of AuNSs

**Fig. 3:**
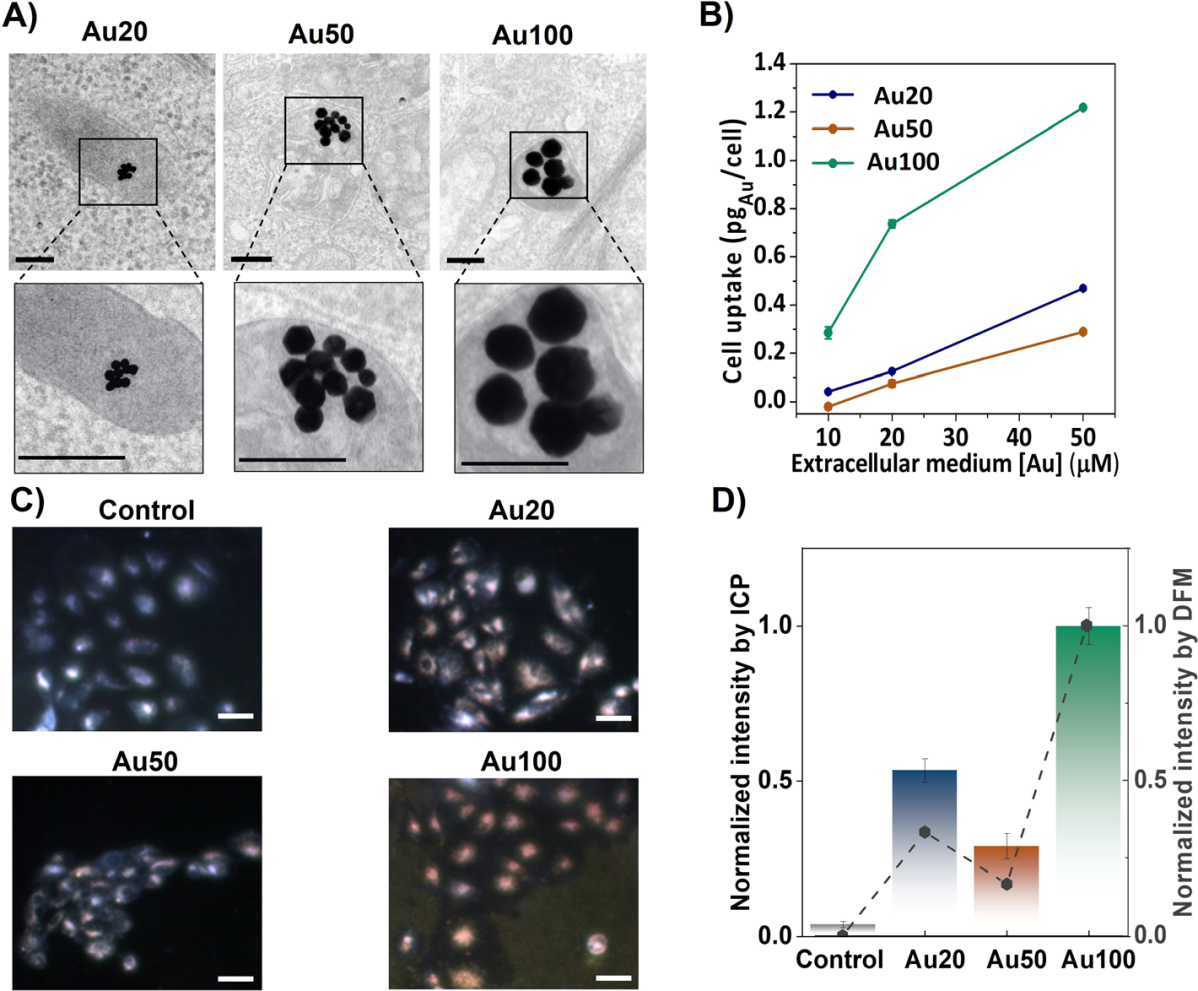
Internalization of AuNSs in cancer cells (MCF-7). (A) Trasmission electron microscopy (TEM) images after internalization of AuNSs with different sizes. All AuNSs can be found confined within endosomes. Bars correspond to 200 nm. The amplified images show the limitation of the endosomes with light grey. (B) Mass of Au per cell after internalization, obtained by ICP-OES measurements, as a function of different extracellular Au concentrations. Cells were digested after removing the media and washing them with PBS. (C) Dark field images of AuNSs in cells. Bars correspond to 10 μm. (D) Normalized DFM mean intensity per cell of the different AuNS sizes (grey dots) compared with normalized cell uptake obtained by ICP-OES.

AuNSs with diameters of 18 ± 2 nm (Au20), 56 ± 8 nm (Au50) and 100 ± 11 nm (Au100), shown in Fig. S2,† were internalized for 24 h into breast cancer cells (MCF-7 cell line) at different extracellular concentrations. After internalization, endosomal confinement of the AuNSs occurs regardless of the size of the AuNSs (see Fig. 3A). Cell uptake increases with extracellular incubation concentrations of AuNSs, resulting in a greater amount of internalized AuNSs. Specifically, for [Au] = 50 μM, the resulting intracellular doses reach a maximum corresponding to 0.4, 0.2 and 1.2 pg_Au_ per cell, for Au20, Au50 and Au100, respectively (see inductively coupled plasma (ICP-OES) measurements, Fig. 3B). For this, the cells were digested after removing the media and washed with PBS (see the ESI†). The presence of AuNSs inside cells was also evaluated *via* dark field microscopy (DFM) images, identifying cells in light blue color and AuNSs as yellowish/reddish dots, as shown in Fig. 3C. This color is a result of the intense LSPR response of the AuNSs in the visible spectral range, which is translated into a higher scattering of light. Using the color-filtering protocol previously reported,^47^ and described in the ESI,† it is possible to isolate and quantify the contribution of the AuNSs per cell from the whole dark field image (Fig. 3D). The low intensity of the control cells (*i.e.*, those that were not incubated with AuNSs), further validates the effectiveness of filtering the contribution of the AuNSs. Fig. 3D also compares the cell uptake extracted from the mean intensity of DFM images with the uptake derived from ICP-OES measurements. Results were normalized to allow the comparison of the tendency of the AuNS uptake with size. Both techniques corroborate a similar dependency on size, showing a smaller uptake for Au50 (normalized values close to 0.25) compared to the uptake obtained for Au20 (values close to 0.5) and Au100 (values close to 1), where the internalization is at its maximum.

### 2.3 Plasmonic response of AuNSs inside cells

**Fig. 4:**
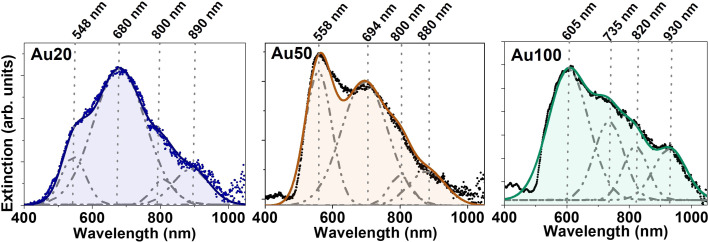
Experimental plasmonic response of AuNSs with different sizes (20, 50 and 100 nm from left to right) internalized in MCF-7 cancer cells. Black dots indicate experimental data. Gaussian deconvolution is represented in dashed grey lines. The colored line indicates the overall fitting of the Gaussian functions with a fit of *R*^2^ > 0.9 in all cases. The identified maxima of the extinction bands are marked with dashed lines. Background contributions from cells have been subtracted from the signal.

To evaluate the effect of internalization of the AuNSs inside the cells, the extinction spectra of MCF-7 cell cultures incubated with different-sized AuNSs were recorded. For these sets of experiments, we adjusted the extracellular concentration to [Au] = 60 μM for 24 h. As AuNSs are internalized in cells, the extinction spectra clearly redshift and broaden, showing the appearance of new coupling modes (compare Fig. 1A and 4). A deconvolution of the spectra of each sample was conducted identifying several resonances. Four extinction bands were obtained for the three different sizes, with a position dependence on the AuNS size and a trend towards longer wavelengths as the size increases. Four bands are the minimum number of optical bands to obtain the highest coefficient of determination, *R*^2^, which determines the good fitting for the three samples. This redshift, followed by the appearance of new bands in the biological window, could affect the photothermal efficiency of these nanostructures for PTT application and their suitability to NIR photoexcitation, allowing effective deep body penetration. For Au20 and Au50, a significant photothermal response is expected in the 550 to 900 nm wavelength range, while Au100 may extend the optical response until 930 nm. All AuNSs being well suited for laser excitation at 680 and 808 nm, the wavelengths employed in this study. The relative intensity and width of the plasmonic resonances also vary depending on the AuNS size, as well as the aggregation state and interparticle distance. All these parameters will affect their photothermal response.

The appearance of new extinction bands in the NIR range during the internalization process suggests the activation of new light–matter interaction processes due, for example, to aggregation and interparticle interactions. To elucidate the mechanisms underlying these new processes, the verification by simulations is required. These new infrared extinction bands could obey an enlarged contribution from scattering or absorption in this spectral range – the sum of scattering and absorption gives the extinction of light.^9^ If the new bands are caused by an increment in the scattering cross-section, the NPs would behave, for example, as good optical probes for cell imaging under infrared illumination. On the other hand, if absorption increases, the NPs inside cells could act as heat transducers. Although there are spectroscopic methods to determine the contributions of each process,^48,49^ simulations can simplify and reduce experimental procedures.^50^ In the photothermal response section, we discuss whether the observed new infrared bands are predominantly caused by an absorption enhancement and, thus, increasing the intracellular heat generation under infrared excitation.

### 2.4 Simulation of the plasmonic coupling of AuNSs inside cells using the FEM model

FEM simulations of AuNSs with different sizes were performed by evaluating several parameters: the type of aggregation of AuNS assemblies, the optical characteristics of the surrounding media of the AuNSs, and the interparticle distance between the AuNSs.

#### (i) Impact of the configuration of AuNS assemblies: types of aggregation arrangements

FEM simulations were performed on different AuNS aggregates, varying the number of AuNSs, mimicking the final size of a representative cluster formed inside endosomes. Indeed, the limited internalization of AuNSs observed within endosomes (Fig. 3A) provides an optimal configuration for simulating a small number of characteristic interacting plasmonic particles. Compact geometries with interparticle distance of 1 nm have been considered for all modeled aggregates. This gap is an intermediate value expected as a consequence of the presence of surfactant molecules attached to the surface of the NPs (0.5–2 nm), which appear as a result of chemical coating during the synthesis process,^51^ or proteins adhered to the surface-forming a corona,^4^ and proper accuracy with meshing and computing time solution. The refractive index (*n*) is chosen to be *n* = 1.6 considering the aggregates within the lysosomes. This *n* value is selected, among those evaluated in this paper, since plasmonic coupling is related to both the AuNS confinement in the localized environment surrounding them and its corresponding value of *n*, as we will discuss in next sections.

**Fig. 5:**
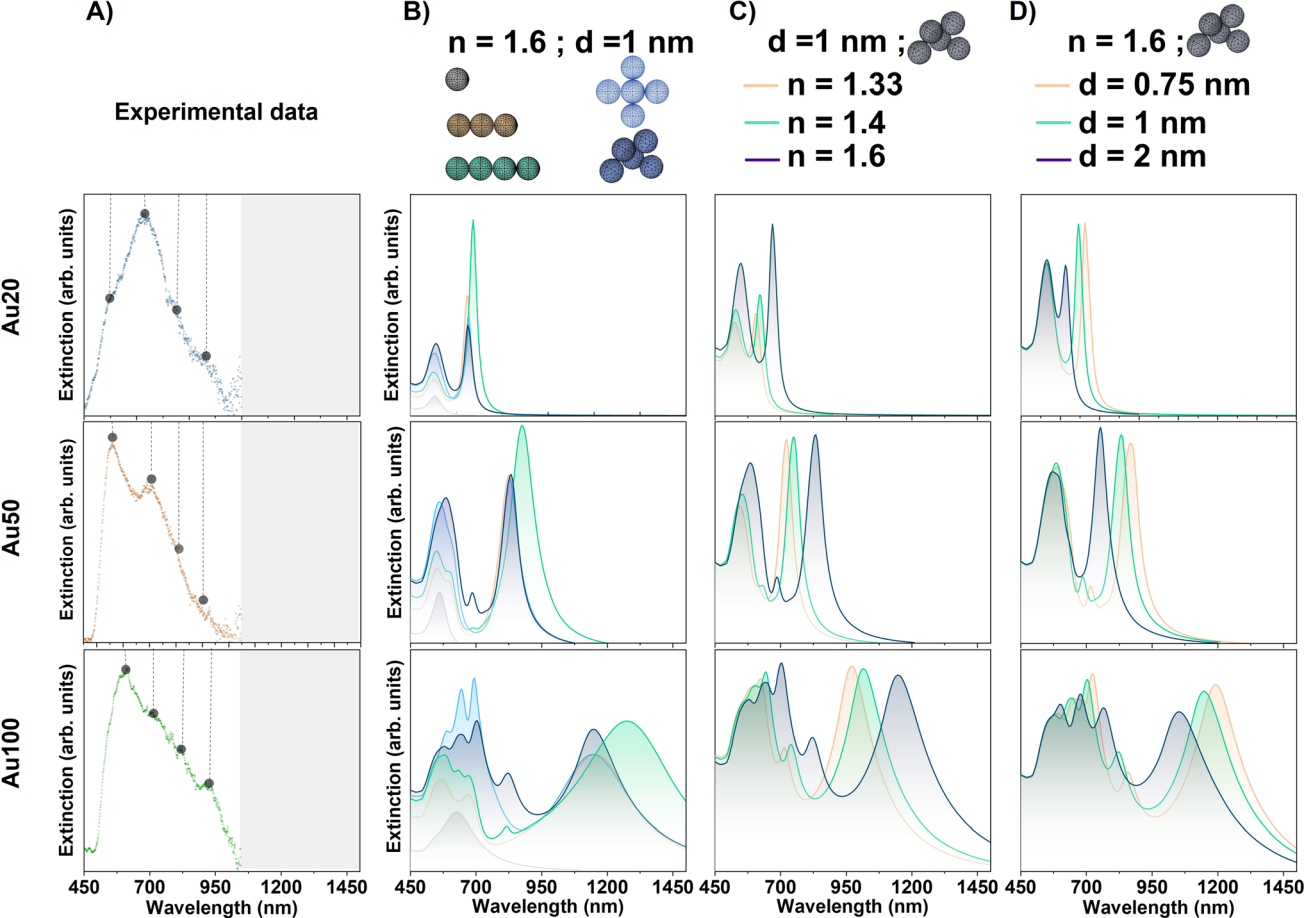
(A) Experimental plasmonic response of AuNSs with different sizes internalized in MCF-7 cancer cells. For better visualization, points mark the maxima of the deconvoluted Gaussian fits in Fig. 4. Grey range of the spectra are experimental data not measured. Evaluation of extinction signal by FEM simulations varying the (B) arrangements of the NS assemblies for Au20, Au50 and Au100, (C) surrounding environment of AuNSs with *n* = 1.33, 1.4 and 1.6 and (D) interparticle gap between AuNSs: *d* = 0.75, 1 and 2 nm. For the different simulations, new resonances varying their position, FHWM and relative intensity are obtained depending on the AuNS size and the conditions considered.

The optical response for Au20, Au50 and Au100 AuNSs is simulated for different aggregate configurations: a single isolated AuNS, 3–4 AuNSs arranged in linear arrays and non-linear aggregates of 5 AuNSs with two different arrangements (ordered and disordered aggregates), as represented in Fig. 5B. For the isolated AuNSs, a single extinction band is obtained in the spectral range at 545.1 ± 0.2 nm, 560.6 ± 0.3 nm and 632.7 ± 0.7 nm for Au20, Au50 and Au100, respectively. This implies a redshift of the monomer contribution from the one calculated in water (Fig. 1) due to the increase of the *n* value of surrounding media.^52^ Remarkably, these spectral bands exhibit proximity to the first band derived from the deconvolution of experimental extinction spectra in Fig. 4 (approximately at 548 nm, 558 nm and 605 nm). This observation can be attributed to the specific localization of the NPs within the lysosomes, thereby being predominantly influenced by this localized environment.^53^ For the rest of AuNS assemblies, not only changes in the position of the resonance are identified. In addition to the appearance of new absorptions due to plasmonic coupling and electromagnetic interaction between the individual AuNSs forming the aggregate, changes in the relative intensity of bands are also found. For linear chain-like aggregates composed of 3 AuNSs, an additional resonance band appears at larger wavelengths, similar to rod-like nanostructures where plasmons are excited in two directions (longitudinal and transverse plasmons).^3^ The major contribution is attributed to the longitudinal direction of the array with the largest redshift and intensity, which is in good agreement with the literature.^3^ Besides, the redshift of the longitudinal plasmon increases with the AuNS size ranging from 667.1 ± 0.3 nm, 826.6 ± 0.5 nm and 1149.5 ± 0.8 nm, for Au20, Au50 and Au100, respectively. A trend can also be defined in terms of the FWHM. As the AuNS size increases, the FWHM increases, extending the range of plasmonic excitation to larger wavelengths. Finally, other extinction bands are identified at lower wavelengths for the largest AuNSs (Au50 and Au100), which can be associated with radiation effects where the movement of electrons is inhomogeneous and multipolar charge distributions are generated.^3^

The symmetry breakage of the AuNS cluster also induces changes in the spectral profile. In addition to the presence of the longitudinal absorption mode identified in the AuNS linear arrays, new resonances appear at lower wavelengths for larger AuNSs identifying a greater intensity of those bands related to the non-linearity of the cluster. In addition, AuNS clusters can support Fano resonances, which depend on the intrinsic and extrinsic parameters such as the cluster geometry, its relative size, interparticle gap and/or surrounding environment.^13,54^ This phenomenon is based on the coupling of the antiparallel dipole modes and can occur in some cluster configurations considered.^13^

These results indicate that, even for the smallest aggregates of Au20 AuNSs, and with a more noticeable effect for larger AuNSs (especially Au100), the impact of the resonant modes on the optical spectrum in the first biological window region (and even the second) is highly suitable for several biological applications. Furthermore, FEM simulations can anticipate the complexity of modes induced by plasmonic coupling due to the aggregation of AuNSs. These simulations show the effect on the vibration bands depending on the AuNS size, with a major number of modes and higher redshift for the largest AuNSs, as it is expected for AuNSs with > 50 nm diameter (see Fig. 5).^55^

It should be mentioned that for these simulations we have not considered very large AuNS aggregates. On the one hand, larger close-packed clusters should not redshift the extinction modes but rather diminish the secondary modes.^56^ On the other hand, an extended array of AuNSs arranged as chains is not considered in this particular case since the average number of AuNSs internalized in endosomes are around 4–5. This arrangement is commonly observed in TEM images as the average configuration along the cell.

In the ensuing sections we have selected the simulated 5-NP non-symmetric aggregate. With this description, we address two different behaviors when NPs are internalized within cells, an associated redshift and symmetry breakage of the modes due to rearrangement of the NPs.

#### (ii) Impact of the optical properties of the surrounding environment of AuNS assemblies

As is well explained in the literature, changing the refractive index of the host media surrounding the dispersed AuNSs shifts the absorption modes. Increasing the *n* value induces a redshift of the plasmonic resonance.^3^ Specifically, when internalizing NPs in cells, one may consider a homogeneous cell *n* as an average of all the contributions given by cells suspended in the immersive medium. Also, each organelle inside the cell has its own *n* value, which is cell line-dependent as well.^39,57^ Employing FEM simulations, we aim not only to quantify the associated redshift when considering critical environments for cells, but also to investigate other induced changes in the absorption spectrum. For that, we run the designed model for three different *n* values: 1.33, 1.4 and 1.6. Firstly, *n* of 1.33 has been chosen taking into account that water, PBS buffer and extracellular fluids have *n* range from 1.33 to 1.36. Secondly, *n* of 1.4 is considered taking into account cell *n* value extracted from the literature, which comprehends values ranging from 1.35 for the immortalized normal oral keratinocyte (INOK) to 1.48 for epithelial cells of the oral cavity.^57^ Specifically, Z. A. Steelman *et al.* measured an *n* value for a single MCF-7 cell of 1.38,^58^ and 1.4 is used as an approximation corresponding also to the typical *n* of skin tissue.^59^ Finally, an *n* value of 1.6 is evaluated to mimic lysosome confinement of AuNSs.^39,60,61^

Fig. 5C shows the extinction signal modeled for Au20, Au50 and Au100 NPs varying the refractive index of the surrounding media of the AuNSs. All simulations are performed considering the non-symmetric 5 AuNS aggregate (simulated in the previous section) with an interparticle distance of 1 nm and no wavelength dependence of the *n* value for cell tissues. For the three different AuNS sizes, an overall redshift of the resonances is observed for the different bands. The displacement depends on the specific resonance itself, resulting in a larger redshift when the band is displayed at higher wavelengths as *n* increases. This observation is in good agreement with the literature.^38^ For example, *n* = 1.6 shifts the extinction maxima to the following higher wavelength values: 670.2 ± 0.8 nm, 831.3 ± 0.7 nm and 1154 ± 1 nm, for Au20, Au50 and Au100, respectively. In addition, modifications in the relative intensity of resonances are noted as the *n* increases. The intensity dependence is expected since the extinction coefficient is proportional to the real permittivity of the medium.^3^

Regarding the simulations that best fit to explain the strong redshift observed in experimental measurements (Fig. 4), the highest value of *n* related to a lysosome environment should be considered for the case of smallest AuNPs (Au20). However, as AuNSs become larger, such a high *n* is not necessary to explain the significant response observed in the NIR-I window. Overall, after evaluating the three different sizes, we conclude that a local confinement of AuNSs in the lysosome environment as a chosen standard *n* value could yield better results for the simulations.

With these findings, and considering the results obtained for different aggregates, one may suspect that a different aggregate configuration may not necessarily require such a high refractive index to shift the modes within the NIR range. However, for this specific situation, the cluster formation should have a significantly large and well displayed chain-like array of AuNSs with small interparticle gaps. This particular configuration was not observed in the TEM images of internalized NPs in cells.

In the following section, we selected a lysosome environment for FEM simulations (*i.e.*, setting up *n* = 1.6) for three different reasons. Firstly, as elucidated in section (i), simulations of isolated particles within this environment can describe the first spectral bands observed for each size of AuNS through the deconvolution of experimental extinction spectra. This can serve as a verification since aggregation can slightly shift the dipolar resonance but must describe this band within the local environment.^53^ Secondly, as we have demonstrated in this section, this particular description gives the best results when comparing to experimental measurements for the three NS sizes. Lastly, the selection of *n* = 1.6 is the most reasonable because of the proper LSPR phenomenon. As NPs are internalized within lysosomes – previously demonstrated by TEM (see Fig. 3A) – local plasmonic interactions may be modulated by the refractive index related to the cell confinement. Local interactions of NPs with the nearest environment, as opposed to interactions with the whole cell, are expected because LSPR is sensitive to local dielectric surroundings, with field decay lengths ranging up to tens of nanometers (usually 5–30 nm) depending on the NP geometry.^62^ Thus, interaction with the lysosome environment will occur, as lysosome size ranges from 200 to 600 nm.^63^

#### (iii) Impact of the interparticle distance of AuNSs

The interparticle gap considered for FEM simulations ranges from 0.75 nm to 2 nm, which determines the AuNP interactions and their plasmonic response. This separation contemplates possible situations such as surfactant molecules adhered during the synthesis^51^ as well as different proteins attached to the surface forming a protein corona, as the AuNPs are immersed in biological media. This protein corona can range from a few nanometers to several tens of nanometers.^64^ Larger gaps were not studied in the simulations, since the optical spectra in that scenario are assumed to recover the optical spectrum profile of an isolated NP.^65^

Fig. 5D displays the modeled extinction signal for Au20, Au50 and Au100 AuNSs varying the interparticle gap distance. For these simulations a 5-AuNS random aggregate with *n* value of lysosomes (*n* = 1.6) is considered. For the three AuNS sizes, the main observed variations are related to a blueshift of the resonances at the longer wavelength bands when the gap size is increased. Varying the interparticle distances from 0.75 to 2 nm, the wavelength variations for the plasmonic resonance at larger positions are approximately 75 nm, 115 nm and 140 nm for Au20, Au50 and Au100, respectively. The shift is associated with an increase of the isolated nature of the NPs, in agreement with the literature.^65^ Additionally, modifications in the relative intensity as well as in the FWHM of the NIR band are observed depending on the interparticle distance. For the extinction band at larger positions, more significant modifications in the relative intensity and FWHM with the gap size between NPs are observed for the largest AuNSs (Au100).

**Fig. 6:**
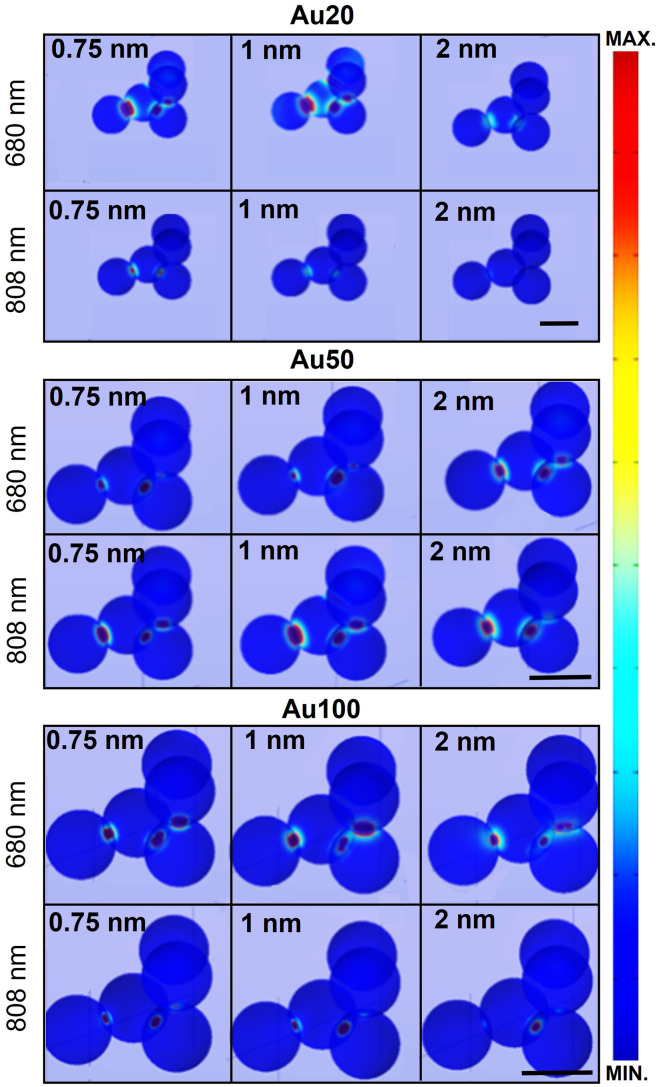
Electric field distribution (*E*/*E*_0_) of the different AuNS sizes at 680 and 808 nm, the two wavelengths used in the photothermal response section. Electric field distribution was determined from theoretical calculation based on FEM using COMSOL Multiphysics. Aggregates of 5 AuNSs have been considered with different interparticle gaps (0.75, 1 and 2 nm). The colour scale corresponds to the electrical field intensity. Hot spots appear within interparticle spacing and their field intensity depends on the AuNS size and gap distance. Scale bars correspond to the diameter of each AuNS.

Fig. 6 displays the simulated electric field diagrams for Au20, Au50 and Au100 AuNSs forming an aggregate with an interparticle distance of 0.75, 1 and 2 nm. The results are provided for the two different wavelengths selected for PTT experiments: 680 and 808 nm. Near contact, an electric field enhancement appears in the surroundings, localized within interparticle gap regions (known as plasmonic hot spots).^66^ The intensity of this enhancement depends on the spacing among the AuNSs, resulting in different coupling enhancements,^67^ as well as on the AuNS size and the excitation wavelength.

The observed field enhancement is related to the extinction spectra of the different AuNS sizes (Fig. 5). From these individual simulations with an evaluation at a specific wavelength, a response is expected for all the AuNS sizes for both selected wavelengths. For instance, the main contribution and field interactions between particles occur for Au20 AuNSs at 680 nm with interparticle gaps 0.75 or 1 nm, and for Au50 AuNSs at 808 nm with a 1 nm spacing.

It should be mentioned that for a thorough analysis of the interparticle spacing, an expected variability must be considered since not all particles will have the same gap between them. Besides that, different configurations on cluster formation will affect the broadening of the bands. However, for an aggregate with more than 5 NPs, the broadening on the absorption signal is not significantly increased.^56^ Other considerations that should be highlighted are the polydispersity on the AuNS size,^68^ which we have observed to drastically change the modes, and the non-ideal (*i.e.*, faceted) AuNSs that exhibit irregular spectral profiles.^69^ These statements can be corroborated through Fig. S4 in the ESI,† displaying the modifications induced in the optical profile increasing the NP number to 6 in the aggregate, varying the distribution of the Au aggregate and using an inhomogeneous interparticle distance (1, 2 and 10 nm) and different NP size (100 and 102 nm).

### 2.5 Photothermal response of AuNSs in cells

**Fig. 7:**
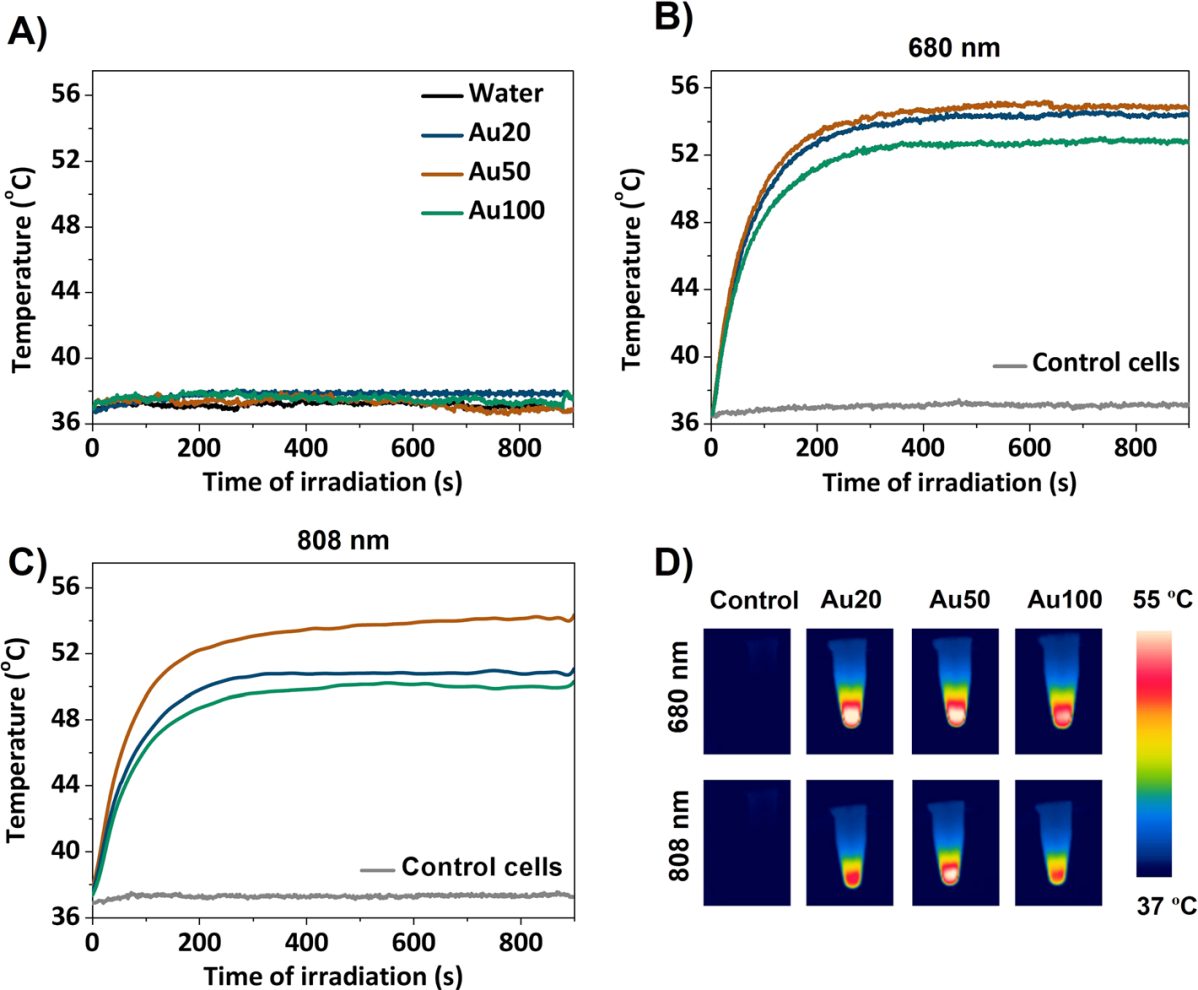
Photothermal response of AuNSs (A) in water, (B) within cancer cells when exposed to 680 nm laser excitation at 0.2 W for 15 min and (C) within cancer cells when exposed to 808 nm laser excitation at 0.2 W for 15 min. (D) Corresponding IR thermal camera recordings for AuNPs in cancer cells under 680 and 808 nm laser excitations.

Photothermal response of AuNSs, varying their size, is investigated in MCF-7 cells at two NIR-I laser excitation wavelengths: 680 nm and 808 nm. The latter is more commonly employed for standard *in vivo* therapeutic photothermal treatments.^2,70^ For that, AuNSs were incubated in cells for 24 h and collected in pellets. Their thermal performance was compared with that of control cells (not incubated with AuNSs) and AuNSs dispersed in water solution. The temperature increase of the samples was monitored and the results are shown in Fig. 7. AuNSs dispersed in water do not exhibit any relevant heat production when illuminated with a 680 nm laser. These results are in accordance with their plasmonic response for the three evaluated sizes of AuNSs as shown in Fig. 1A. In this figure, AuNSs exhibited a plasmon resonance centered in the range of 520–575 nm. As an example, results at 680 nm are shown in Fig. 7A. In all cases, the temperature increase was less than 1 °C compared to a control solution with just water. After internalization of AuNSs in the cells, light to heat conversion in the NIR range clearly becomes efficient. After a few minutes, a plateau temperature is reached, with an increased temperature of 19 °C for Au20 and Au50, and 17 °C for Au100 under 680 nm irradiation (Fig. 7B). For 808 nm laser excitation, the behavior is similar, with an increase of approximately 17 °C for Au50 and 14 °C for Au20 and Au100 (Fig. 7C). Fig. 7D displays the corresponding infrared (IR) camera recordings for different AuNSs in cancer cells under both 680 and 808 nm laser excitations. All evaluated AuNSs in cells can exhibit high light-to-heat conversion when illuminated at an appropriate wavelength, in accordance with the LSPR positions found in both experimental and simulated data (see plasmon response in Fig. 4 and 5, respectively).

**Fig. 8:**
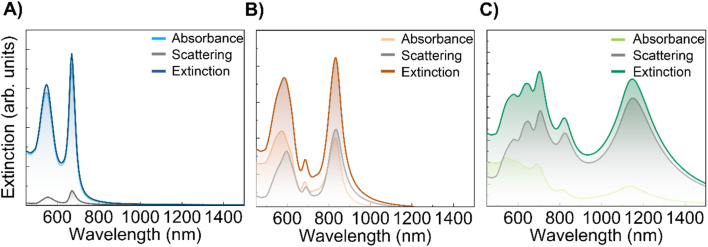
FEM simulations of absorption, scattering and extinction contributions for a 5 AuNS randomly positioned aggregate, with an interparticle gap of 1 nm and lysosome environment for (A) Au20, (B) Au50, and (C) Au100.

Experimentally, the extinction bands of all the AuNS sizes show response in the NIR range up to 1000 nm, predicting that AuNSs could act as great photothermal agents in the NIR range once the nanomaterials are internalized into cells. Regarding FEM simulations, almost all resonance bands are simulated. Nevertheless, for Au20 NSs, the spectrum contributions found experimentally at 890 nm (Fig. 1) could not be reproduced by our simulations. As previously explained, for smaller AuNSs a large widening of the bands is expected, and the contributions experimentally observed at 890 nm may be related to the broadened bands at around 700 nm found by specific simulations. On the other hand, the fact that Au50 and Au20 AuNSs heat more efficiently than Au100, even though resonance bands are within the NIR-I range, could be due to a higher contribution of absorption to the extinction response. As it is mentioned above, heat production is attributed to absorbed photons; however, larger AuNSs have dominant scattering effects, limiting their efficacy as photothermal agents.^9^ Interestingly, even the smallest AuNSs (Au20), which display in water their LSPR at shortest wavelengths (approximately 525 nm), show great photothermal response under NIR light at 808 nm after internalization, assuming therefore mainly contributions of absorption within the aggregates for this NS size. Simulations predict both behaviors, expecting better absorption and heat generation when analyzing smaller NPs. The simulations in Fig. 8 were performed to analyze absorbance and scattering contributions in a lysosome environment for AuNSs of three diameters with a 1 nm interparticle gap. For Au20, absorbance is the main contributor to extinction, suggesting enhanced photothermal processes in the NIR range during internalization. Larger NPs exhibit increased scattering contributions, according to the literature.^3^ These simulations explain the reduced photothermal response of Au100 compared to Au20 and Au50, despite sufficient absorption for heat generation.

**Fig. 9:**
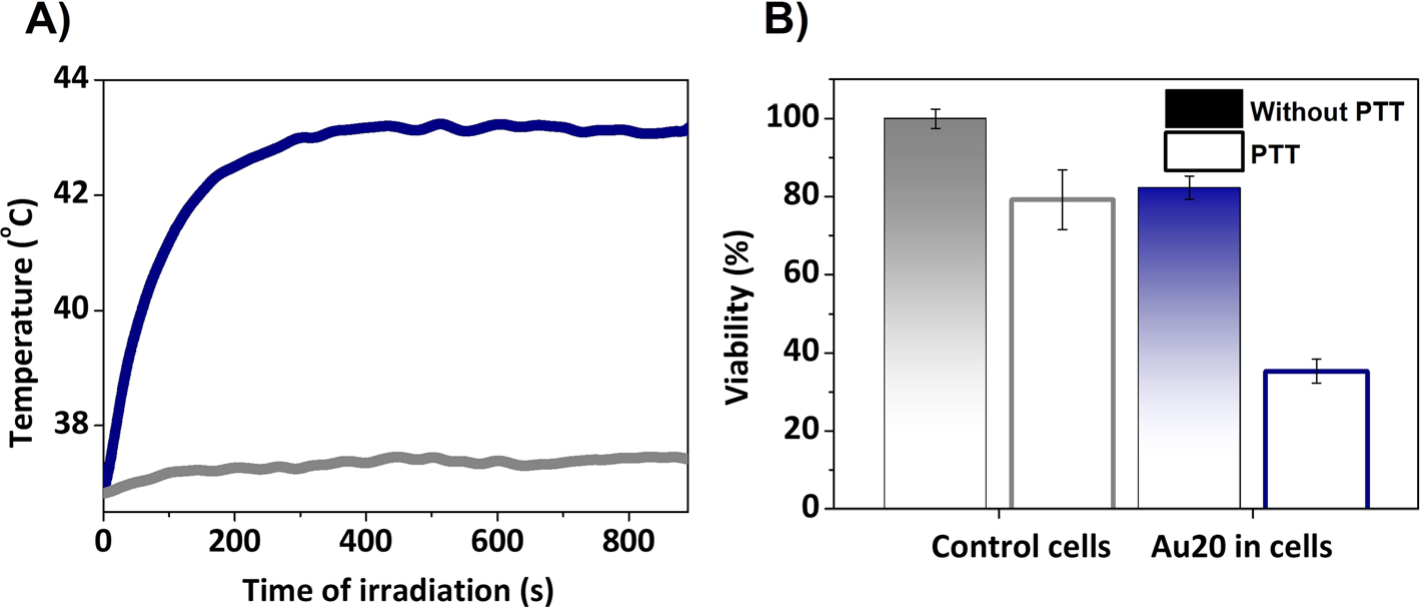
(A) Photothermal response of Au20 NPs under 808 nm laser excitation at 0.2 W for 15 min. (B) Viability test assessed 24 h after PTT.

Finally, by regulating the laser power irradiation, well-controlled parameters can be attained to facilitate an effective photothermal treatment. In this study, a power density of 0.17 W cm^−2^ was used and increments of temperature of almost 6 °C were achieved (Fig. 9A), which correspond to a temperature treatment of 43 °C. This is in good agreement with the standard macroscopic temperature range for hyperthermia therapy, which is typically between 41 and 45 °C to prevent irreversible damage and unwanted injuries. In this case, the power laser radiation is within the safe clinical conditions (0.33–1 W cm^−2^).^71^

In order to correlate the temperature achieved with cellular damage, cell survival tests were conducted 24 h after the irradiation. For this, the cells were incubated with AuNSs, trypsinized and collected in pellets, and finally irradiated with a 808 nm laser (PTT) or untreated. Control cells without AuNSs were compared to cell pellets with AuNSs, both with and without PTT. 24 h after PTT, the viability of the cells incubated with AuNSs was reduced and only 35% survived (Fig. 9B), compared to a viability superior to 80% in the culture with AuNSs that was not irradiated. The latter value differs from the initial cytotoxicity assays due to variations in cell adhesion during detachment and reseeding (see Fig. 8). In summary, this indicates the effectiveness of the treatment in cells. Moreover, cell susceptibility to irradiation and other treatments such as chemotherapy or radiotherapy, is expected.^72^

Therefore, the internalization of AuNSs by cells has led to an efficient conversion of laser irradiation into heat, facilitated by a broad plasmonic band in the NIR range. These results are consistent with the experimentally observed and simulated LSPR positions performed in the previous sections. These findings align with previous studies demonstrating enhanced photothermal response in cells with AuNPs sized between 10 and 25 nm,^8,17,18^ despite their original suboptimal plasmonic properties.

## 3 Conclusions

In this study, the plasmonic behavior of gold nanospheres (AuNSs) of different sizes has been investigated by creating a 3D model based on the finite element method in COMSOL Multiphysics. This model is validated for colloidal stable AuNSs in an aqueous medium, both through experimental analysis and with the Mie theory. By considering the aggregation of the AuNSs when interacting with biological media, the FEM model provides an explanation for the observed experimental results. The key parameters influencing the AuNS stability and inducing the plasmonic coupling have been identified, such as the different types of aggregation arrangements, the optical properties of the surrounding environment or the interparticle distance. All these conditions have a critical impact on the characteristics of the spectral bands, and the appearance of new resonances, all of which have implications for the AuNS photothermal response, and can be then anticipated to design more effective photothermal treatments. Experimental and theoretical techniques play a vital role in understanding the underlying physical mechanisms in complex systems such as a tumor microenvironment.

## Data availability

The data that support the findings of this study are available from the corresponding authors upon reasonable request.

## Author contributions

The manuscript was written through contributions of all authors. All authors have given approval to the final version of the manuscript. Marina París Ogáyar: writing – original draft; software; data curation; formal analysis; investigation. Rosalía López Méndez: review & editing; data curation; formal analysis; investigation. Ignacio Figueruelo-Campanero: review & editing; software. Tamara Muñoz-Ortiz: writing/review & editing; data curation. Claire Wilhelm: writing/review & editing; methodology. Daniel Jaque: writing/review & editing; resources; methodology. Ana Espinosa: writing – original draft; conceptualization; supervision; resources; formal analysis; funding acquisition; investigation; methodology. Aida Serrano: writing – original draft; conceptualization; supervision; resources, methodology; formal analysis; software; funding acquisition.

## Conflicts of interest

The authors declare no competing financial interest.

## Supplementary Material

NA-006-D4NA00247D-s001
