# Supplementary material for: Finite element modeling of plasmonic resonances in photothermal gold nanoparticles embedded in cells
Source: Nanoscale Adv. 2024 Jul 10;6(18):4635–46. doi: 10.1039/d4na00247d (PMC11386126; doi:10.1039/d4na00247d)
Supplement: NA-006-D4NA00247D-s001 [file NA-006-D4NA00247D-s001.pdf]

Supplementary Information (SI) for Nanoscale Advances.
This journal is © The Royal Society of Chemistry 2024

Supporting information

Finite element modeling of plasmonic resonances in photothermal gold nanoparticles embedded in cells

Marina París Ogáyar[1,2,3], Rosalía López Méndez[2], Ignacio Figueruelo-Campanero[1,2,4], Tamara Muñoz-Ortiz[3,5], Claire Wilhelm[6], Daniel Jaque[3,7], Ana Espinosa[8,2,*], Aida Serrano[1,*]

[1] Instituto de Cerámica y Vidrio, CSIC, C/Kelsen, 5, 28049, Madrid, Spain
[2] IMDEA Nanociencia, C/Faraday, 9, 28049, Madrid, Spain
[3] Nanomaterials for bioimaging group (nanoBIG), Departamento de Física de Materiales–Facultad de Ciencias, C/ Francisco Tomás y Valiente, 7, Universidad Autónoma de Madrid, 28049, Madrid, Spain
[4] Departamento de Física de Materiales, Universidad Complutense de Madrid, Plaza Ciencias, 1, 28040, Madrid, Spain
[5] Instituto Nicolás Cabrera, Facultad de Ciencias, C/ Francisco Tomás y Valiente, 7, Universidad Autónoma de Madrid, 28049, Madrid, Spain
[6] Laboratoire Physico Chimie Curie, PCC, CNRS UMR168, Institut Curie, Sorbonne University, PSL University, Paris, 75005 France
[7] Institute for Advanced Research in Chemical Sciences (IAdChem), C/Francisco Tomás y Valiente, 7, Universidad Autónoma de Madrid, Madrid 28049, Spain
[8] Instituto de Ciencia de Materiales de Madrid (ICMM-CSIC), C/ Sor Juana Inés de la Cruz 3, 28049, Madrid, Spain

Table of contents

(I) Materials and methods

(I.I) Materials

(I.II) Methods

(a) Cell culture and cellular uptake

(b) Transmission electron microscopy

(c) Dark field microscopy

(d) Plasmonic response

(e) Plasmonic simulations

(f) Thermal measurements

(S1) Schematic of experimental setup

(II) Results

(S2) Transmission electron microscopy

(S3) Viability assay

(S4) Plasmonic simulations

(III) References

(I) Materials and methods

(I.I) Materials

Commercial AuNSs in citrate buffer stabilized suspension (Sigma-Aldrich) of different diameters 20, 50 and 100 nm were employed. NSs are denoted in accordance with the diameter Au20, Au50 and Au100 (see **Figure S1**).

(I.II) Methods

(a) Cell culture and cellular uptake

For *in vitro* measurements, breast cancer cells (MCF-7 cells) were cultured in Dulbecco's modified Eagle's medium (DMEM) supplemented with 10% fetal bovine serum (FBS), 1% penicillin and L-glucose and maintained at 37 °C with 5% CO_2 until confluence.

Cell viability essays using AlamarBlue were performed increasing extracellular concentration, [Au] = 10, 20 and 50 µM. Fluorometric readings were measured in a microplate reader Lector Biotek with 530 nm excitation and 590 nm emission (Figure S2). The reagent was used as background control.

Cellular uptake was evaluated by inductively coupled plasma optical emission spectroscopy (ICP-OES) in a Perkin Elmer Optima 2100 DV apparatus (ICMM-CSIC, Spain). For this, AuNSs were incubated increasing extracellular concentration [Au] = 10, 20 and 50 µM. After removing the media, cells were washed with PBS to eliminate the non-internalized NPs. Subsequently, cells were harvested after 24 h and resuspended in aqua regia in 1.5 mL Eppendorfs. Samples were heated up in a thermoblock for 2 h at 80 °C and finally diluted in 10 mL Milli-Q water.

(b) Transmission electron microscopy

For cell images, cells labeled AuNSs were incubated at [Au] = 60 µM for 24 h and fixed with a suspension of 4% paraformaldehyde (PFA) and 2% glutaraldehyde in 0.1 mol/L sodium cacodylate buffer for 2 h, and postfixation in 1% *osmium tetroxide*, *containing 1.5% potassium cyanoferrate*, and embedding in resin and cut in sections with microtome. The images were recorded with a JEM1400 Flash (Jeol) (CBM-CSIC, Spain).

(c) Dark field microscopy

MCF-7 cells were incubated with AuNSs in DMEM dispersion adjusting extracellular concentration to [Au] = 60 µM for 24 h. For DFM imaging, a Nikon Eclipse Ti2-U microscope with a condenser (numerical aperture, NA, of 0.80-0.95) and a 20× objective (NA =0.40) was employed. Cells were illuminated with a broadband white LED source and images were taken with a visible CMOS camera from UEye, Thorlabs**.**

The contribution of the AuNSs to the whole scattering signal in DFM images, and hence, their internalization in cells was evaluated by applying a color filter protocol previously reported to the raw images[1]. For that purpose, a custom MATLAB code (version R2022b) was created using the Color Thresholder app. The color channels were threshold in the HSV color space to filter only those yellow-red pixels that are related to the AuNSs. Therefore, blue pixels of the images (corresponding to cells) are removed, isolating the contribution of the AuNSs to the image. After that, using ImageJ software, regions of interest were defined for 30 cells for each NS size under study, and the intensity per cell due to the NSs was determined. Finally, the mean intensity per cell was obtained and the uncertainty was defined as the standard error (i.e., the standard deviation normalized by the root square of the number of cells).

(d) Plasmonic response

Optical extinction measurements were assessed with a real-time UV-Vis Varian 50 spectrophotometer. For AuNSs cell plasmonic characterization, T75 flasks were prepared for each NS size. AuNSs were incubated for 24 h with DMEM adjusting extracellular concentration to [Au] = 60 µM. After 24 h, cells were washed thoroughly and harvested. After that, a lysis procedure was implemented to improve signal-to-noise ratio. For that, after harvesting cells, 600 µL of hypotonic lysis buffer (HB) at 4 °C was added and mechanical cell disruption was carried out with a needle several times to extract the endosomes. To acknowledge that AuNSs in HB media at high concentrations do not affect the aggregation state, UV-Vis measurements were carried out at 0 h and after 2 h of dispersing the AuNSs in HB.

(e) Plasmonic simulations

Physical parameters such as the surrounding media, the interparticle distance and the aggregation state of AuNSs in cells were evaluated with a finite element method (FEM) analysis in the radiofrequency (RF) module of COMSOL Multiphysics 5.3. A 3D model wizard was developed, solving the frequency domain form of Maxwell's equations which is written as **Equation 1**:

$$\nabla \times \left(\mu_r^{-1} \nabla \times E\right) - k_o^2 \left(\varepsilon_r - j\frac{\sigma}{\omega \varepsilon_o}\right) E = 0 \qquad [1]$$

The model consists of a sphere with variable radio surrounded by a perfectly matched layer (PML), truncating the computational domain.

Scattering and absorption cross-sections were evaluated by integrating the scattered Poynting flux vector (S_{sca}) over the surface of the NP and by calculating the volume integral of the power dissipation (Q) inside the NP.
The validation of the 3D FEM model and the plasmonic response of AuNSs of different diameters was carried out for nanoparticles dispersed in aqueous media using MiePLot v4.6.14 program simulation. For the modeling, the distribution of AuNSs was considered using the data obtained by Image J program from TEM images.

(f) Thermal measurements

Thermal measurements of dispersed AuNSs in aqueous solution and confined within cells were both performed in 0.5 mL Eppendorf tubes with a 40 µL sample. For dispersed NPs in aqueous solution, concentration of Au was calculated to be the same as those in cells after cellular uptake consideration. For cells, a T75 flask of confluent cells incubated for each AuNS size was prepared (~ 8×10^6 cells). AuNSs were incubated with DMEM adjusting extracellular concentration to [Au] = 60 µM for 24 h. The cells were then trypsinized and divided into two populations to create irradiated and non-irradiated conditions (~ 4×10^6 cells). For each condition, the cells were collected in a 0.5 mL Eppendorf tube with approximately 40 µL of volume, conforming cell pellets of ~ 4×10^6 cells each. For PTT, samples were illuminated using a diode laser system with two different wavelength excitations (680 and 808 nm) coupled to an optic fiber and SMA fiber collimator at 0.2 W imaged with an infrared camera (FLIR systems). PTT experiments were performed for 15 min of laser excitation and at low power density 0.2 W. The irradiation was performed by illuminating the sample at the top of the Eppendorf tube and the infrared camera was placed at 90° (**Figure S1**).

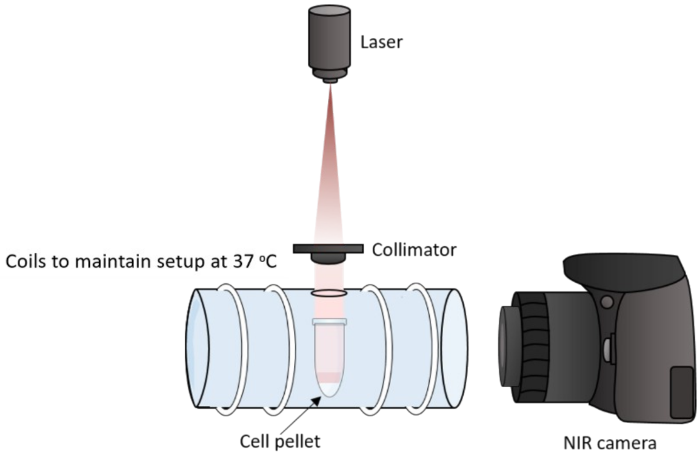

Figure S1. Schematic of the photothermal experimental setup.

(II) Results

(S2) Transmission electron microscopy

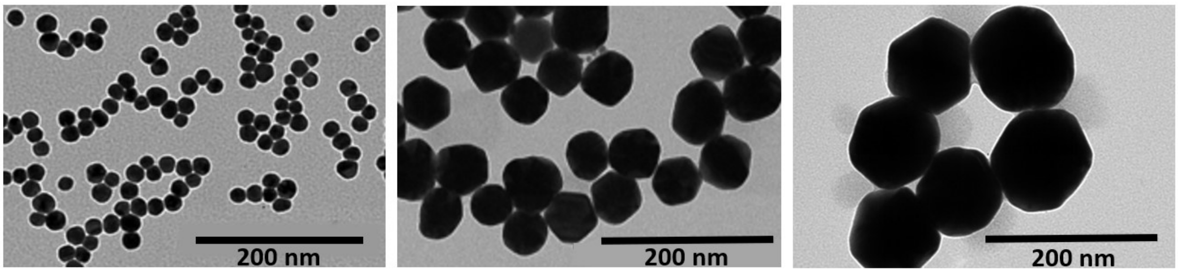

Figure S2. TEM images of Au20, Au 50 and Au100 from left to right.

(S3) Viability assay

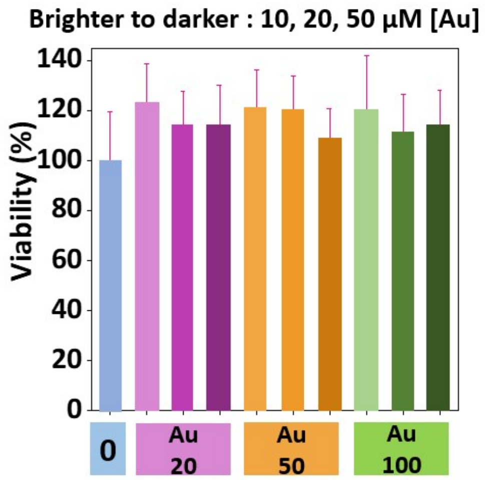

Figure S3. Viability assay varying AuNSs extracellular concentration [Au] = 10, 20 and 50 µM for the three AuNS sizes.

(S4) Plasmonic simulations

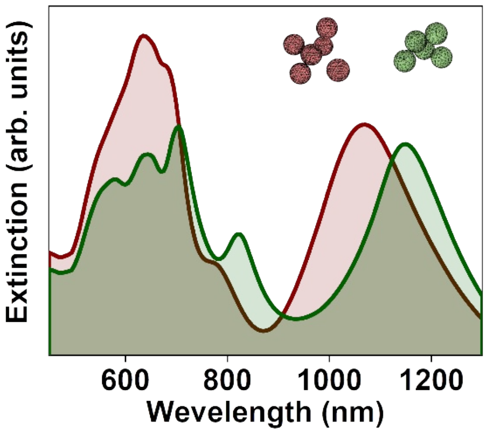

Figure S4. Evaluation of extinction signal by FEM simulations varying the arrangements of the NSs assemblies for Au100. Green signal corresponds to an aggregate of 5 Au NPs with n = 1.6 and interparticle distance of 1 nm. Red signal is related to an aggregate of 6 Au NPs with another distribution, varying the distance between NPs (1, 2 and 10 nm) and the size of NPs (100 and 102 nm).

(III) References

1 T. Muñoz-Ortiz, J. Hu, F. Sanz-Rodríguez, D. H. Ortgies, D. Jaque, D. Méndez-González, R. Aguilar, F. Alfonso, F. Rivero, E. Martín Rodríguez and J. García Solé, *Nanomedicine: Nanotechnology, Biology, and Medicine* 43 (2022) 102556, DOI:10.1016/j.nano.2022.102556.
